# Diagnosis, Management, and Prognosis of Epithelioid Hemangioendothelioma of Maxillary Sinus: A Case Report

**DOI:** 10.7759/cureus.35692

**Published:** 2023-03-02

**Authors:** Arjavon T Talebzadeh, Nojan Talebzadeh

**Affiliations:** 1 Surgery, California Northstate University College of Medicine, Sacramento, USA; 2 Surgery, South County Surgery Center, San Diego, USA

**Keywords:** sarcoma, sinus, epithelioid hemangioendothelioma, tumor, maxilla

## Abstract

Epithelioid hemangioendothelioma (EHE) is a rare condition arising from endothelial cells of the blood vessels. This is a vascular tumor that may occur anywhere throughout the body. This tumor behaves on a spectrum as either a benign tumor or an aggressive sarcoma. The EHE tumor and its management depend on the location of the lesion and accessibility for surgical excision. This case is a rare example of a patient presenting with a maxillary aggressive EHE tumor. This lesion was incidentally found on head CT done for ruling out fractures of the mid-face with incidental findings of an asymptomatic destructive lytic lesion. The treatment of this tumor located in a vital region of the mid-face will be discussed.

## Introduction

Epithelioid hemangioendothelioma (EHE) is a rare vascular neoplasm with incidence of 0.038/100000 per year [1,2]. The incidence of EHE peaks during the fourth and fifth decades [3]. Epithelioid hemangioendothelioma can occur anywhere in the body as either a single lesion or a lesion with local to systemic metastases [4]. It most commonly presents within the soft tissues, bone, liver, or lungs [5].

The presentation of EHE varies from asymptomatic and localized to systemic complications based on the area of involvement. Thus, EHE is often an incidental finding on CT or MRI. Possible symptoms of EHE include pain, mass effect, fever, or weight loss. At times, these patients can also present with venous obstruction in a specific organ or region leading to the diagnosis. Up to half of the cases of EHE arise from or are associated with a vein whereas, in the rest of the cases, recurring symptoms of edema or thrombophlebitis can occur due to venous occlusion facilitating the diagnosis of EHE. Additionally, undiagnosed anemia has been identified in multiple patients with EHE.

Being a rare condition, consistent studies haven't been made to guide diagnosis and treatments. Suboptimal planning and treatments have affected patient outcomes and an attempt has been made to further study the best treatment options. Most findings have been associated with anecdotal cases, and no phase II or III trials have been conducted for EHE treatment. There are two ongoing prospective studies for the two medications: trametinib and eribulin [6,7]. There is limited data available to effectively assess the prognosis of EHE. The recent recommendation includes sequential imaging to monitor growth and tumor aggressiveness over time, which is crucial to direct the treatment plan [8].

## Case presentation

A 66-year-old female presented to the emergency room with abdominal pain. She was treated conservatively and sent home to follow up with her primary care physician. She experienced constipation while using the restroom and suffered a syncopal episode. This led to the loss of consciousness, a fall, and a head injury. She was subsequently transported to the emergency room for re-evaluation. En route, emergency medical technicians identified a systolic blood pressure in the 70s and administered intravenous fluids, Zofran, and checked her serum glucose levels. Her random serum glucose was 154 mg/dL at the scene.

In the emergency room, she complained of mild right posterior parietal headache. She denied shortness of breath and any chest pain or discomfort. She also denied a history of blood in stool or bleeding history. Her medical history was significant for hypertension and asthma, and she had a history of pelvic mass excision three years ago which was benign. Her medications at the time of presentation included albuterol, calcium docusate, ferrous sulfate, losartan, and pantoprazole. She had a negative smoking history and an allergy to shrimp.

Her blood pressure had stabilized at 125/59 with a normal heart rate, oxygen saturation on room air, and temperature. Her head, ears, eyes, nose, and throat (HEENT) exam was significant for tenderness over the right posterior parietal area of the scalp. Her right orbit was tender to palpation and she had some peri-orbital swelling. Her extraocular motions were intact and there was no entrapment of her orbital muscles. Abdominal and neurologic exams were unremarkable with normal bowel sounds. The rectal exam was negative for blood, and the stool appeared normal. Her chemistry was significant for sodium of 135 mmol/L, and her complete blood count (CBC) was notable for hemoglobin of 8 g/dL and hematocrit of 24.6. Chest X-ray was unremarkable. Left ventricular hypertrophy was noted on a 12-lead ECG.

**Figure 1:**
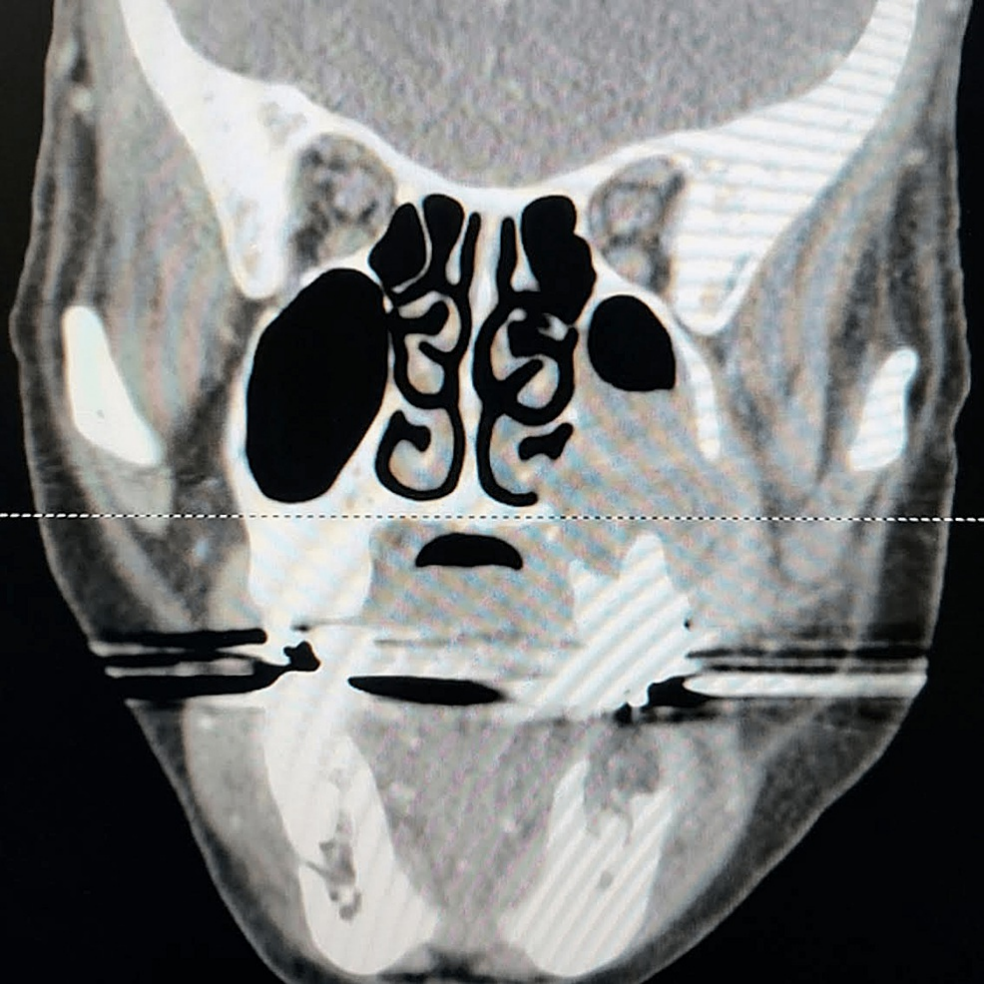
Coronal view of the left maxillary sinus is significant for mass destruction of the lateral sinus wall extending to the left maxillary vestibule and the medial maxillary wall extending to the nasal floor

**Figure 2:**
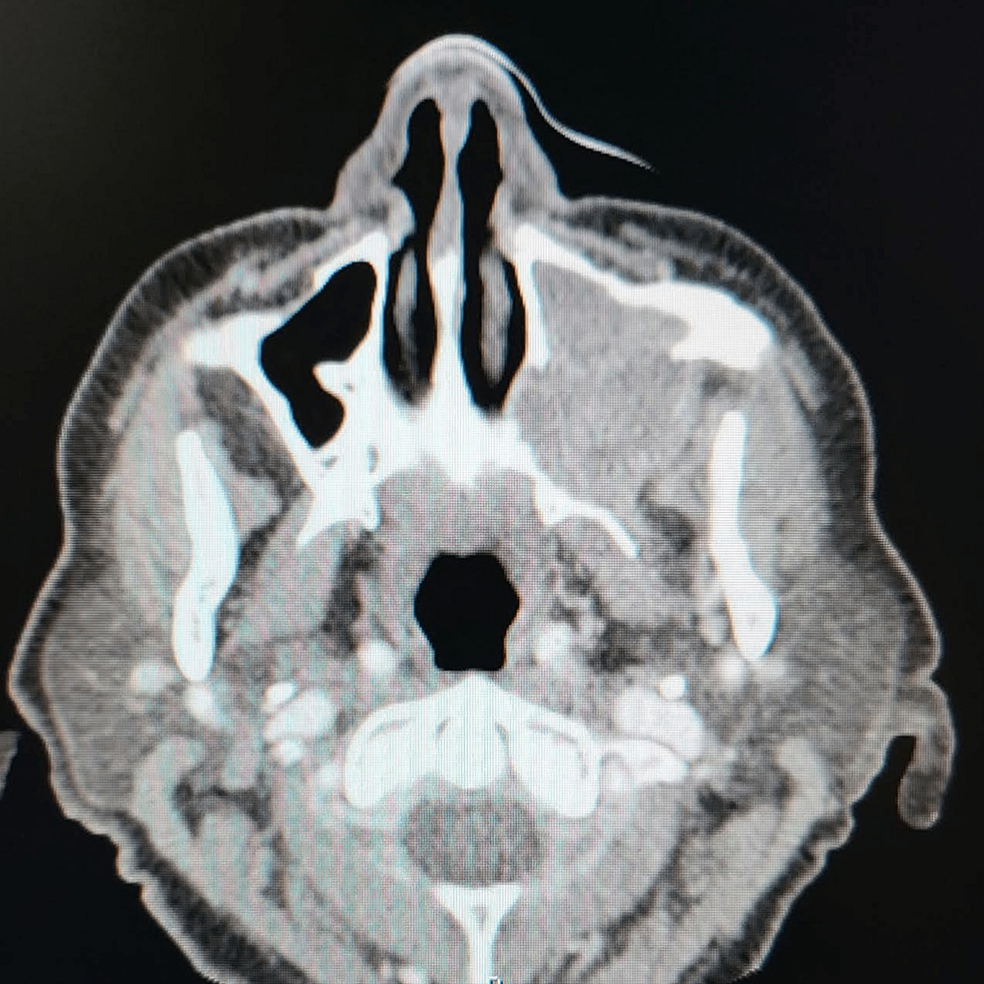
This view reveals the extension of the lesion to the medial and lateral walls of the maxillary sinus. Also, its extension to the pterygoid plates is noticeable.

**Figure 3:**
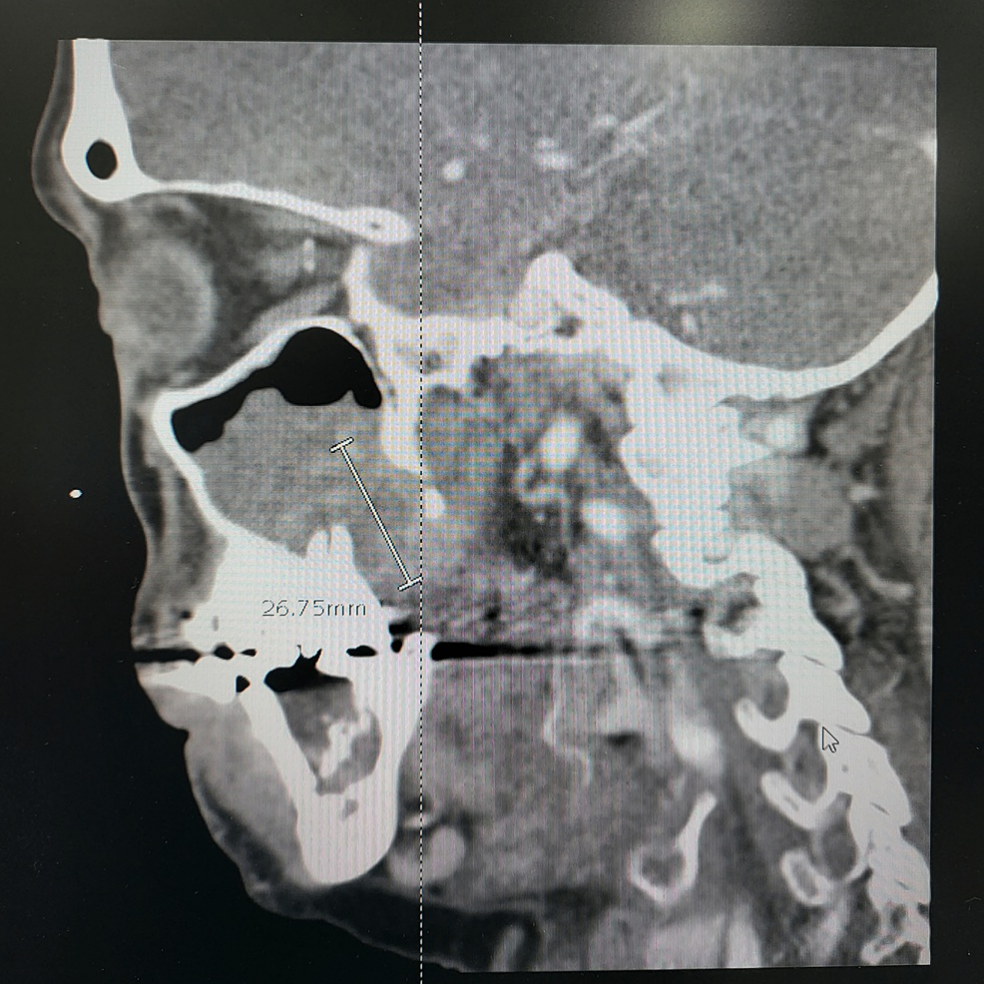
Sagittal CT shows the extent of the lesion. The lesion in this view extends to the pterygoid plates posteriorly.

A head CT scan showed no acute abnormality and a probable left orbital floor fracture with blood in the maxillary sinus. A facial CT also had an incidental finding of a destructive lesion, which was concerning for osteomyelitis versus malignancy (Figures 1, 2, 3). The CT scan also demonstrated a possible lesion in the left mandibular body with positive lymphadenopathy of the left cervical nodes. 

**Figure 4:**
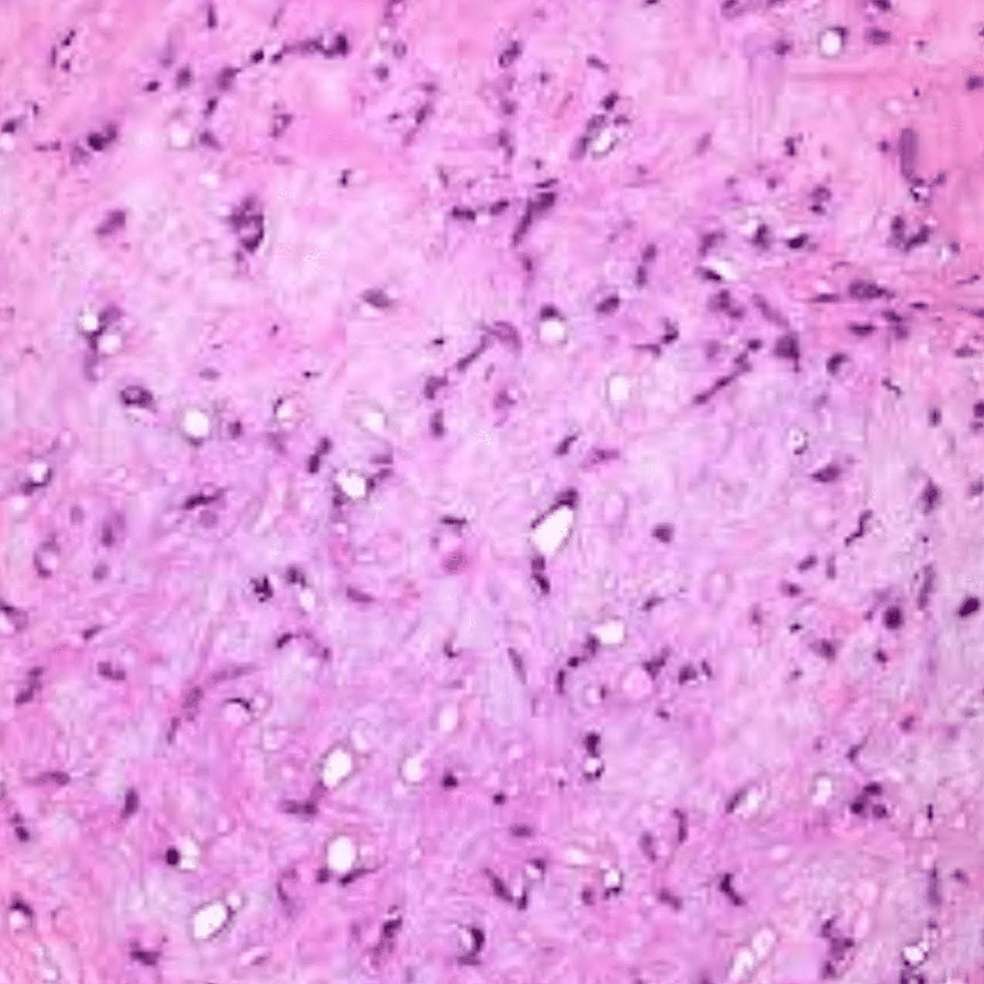
Poorly defined vascular channels mixed with a hyaline stromal matrix and intracytoplasmic vacuoles are seen combined with the myxoid stroma, and the epitheloid eosinophilic cells are seen in the background

**Figure 5:**
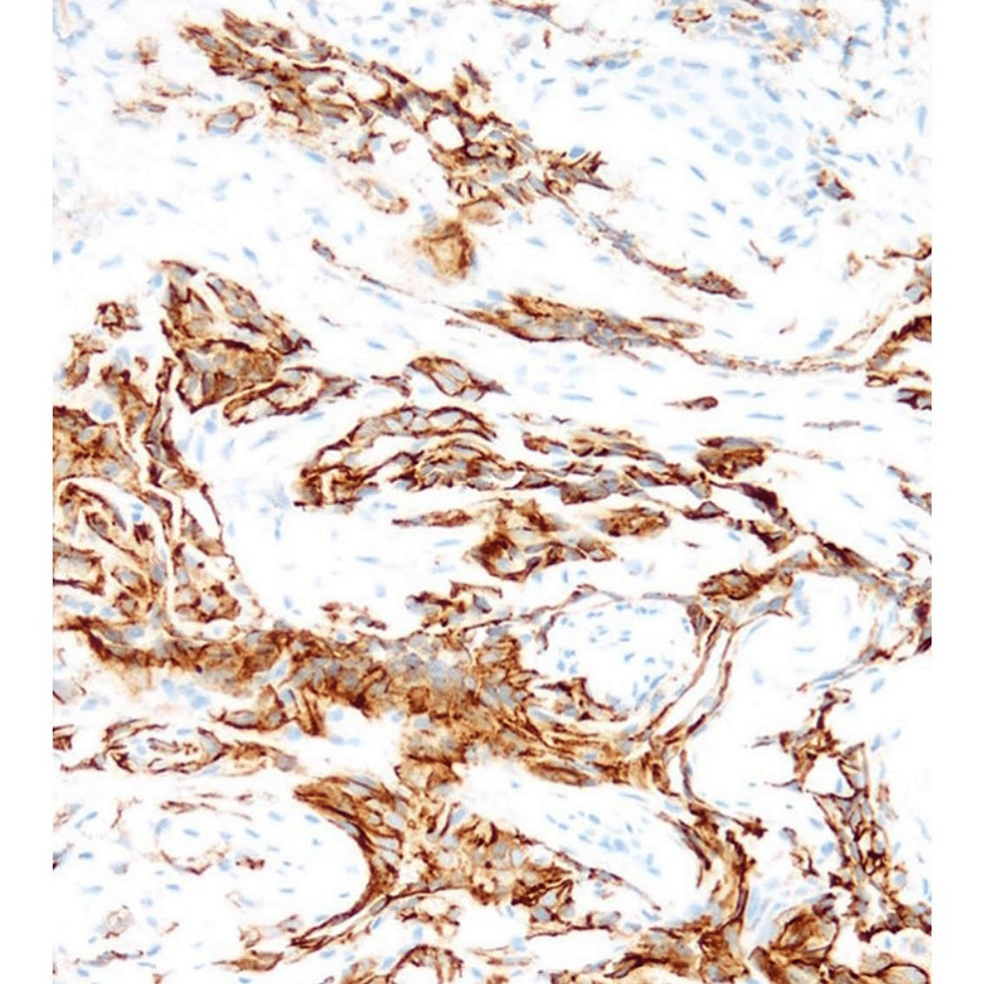
Immunoperoxidase stain confirmation of epithelioid hemangioendothelioma

We were consulted for evaluation. She was taken to the operating room and a left Caldwell-Luc procedure with a biopsy of the intra-sinus mass was carried out. The mass was excised and debrided as much as surgical access allowed (Figure 4). The result of the biopsy after the immunoperoxidase panel was compatible with EHE (Figure 5).

The excision of the mass was done in combination with bone debridement. No margins could be obtained posteriorly and medially due to severe proximity to multiple vital structures including the posterior superior alveolar artery, inferior orbital artery, and branches of the trigeminal nerve. Once the patient was stabilized, she was discharged to be treated for this lesion after a full body workup and consideration for sequential imaging. A full body Scan was obtained with negative metastatic findings. On follow-up with oncology, the decision was made to observe the lesion with serial scans and if necessary in the future to provide localized radiation to minimize injury to adjacent structures. 

## Discussion

Epithelioid hemangioendothelioma is an extremely rare vascular condition affecting the epithelial cell lining of blood vessels. The tendency for this disease to behave as either a mild low-grade growing tumor to a severely aggressive disease. Presentations of EHE range from asymptomatic to severe pain (i.e., abdominal pain, bone pain, or dry cough) and malaise with the presentation of metastatic cancer. There have been cases of skin involvement which has been confused with other pathological entities. 

Epithelioid hemangioendothelioma is not a heritable condition, but some genetic changes have been associated with this condition. These include products of genetic translocations such as yes-associated protein1 (YAP1)-transcription factor E3 (TFE3) gene fusions or WW domain-containing transcription regulator 1 (WWTR1)-calmodulin binding transcription activator 1 (CAMTA1). There has been no consensus on treatment until the European Society for Medical Oncology (ESMO) in 2020 attempted to align patient treatments and provide guidelines for treatment and future research for EHE [9]. Based on the 2020 ESMO recommendations, immunohistochemical or molecular assessment of WWTR1-CAMTA1 and/or YAP1-TFE3 may be necessary. Histologically this lesion appears as epithelioid endothelial cells in myxohyaline stroma. Upon confirmation of this histology, whole-body imaging including CT or MRI or both should be carried out to detect trunk or limb pathology. 

Treatment of EHE patients depends upon the location and spread of the disease. Surgery is the treatment of choice for localized accessible lesions. Excision with a small margin and following the tract of needle biopsy is the ideal treatment where a cure rate of 70% to 80% is expected with negative margins [10]. In cases where complete excision is not possible, other combination modalities including surgery, radiation, other ablative procedures, or even isolated limb perfusions may be indicated. Limited research is available as to the effectiveness of radiation on EHE. It has been documented that EHE can be sensitive to radiation therapy. A total dose of 60 Gy in fractionated fashion is recommended [11]. Currently, there is no definitive study for systemic treatment in patients with metastatic disease. Interferon, thalidomide, and rapamycin are subjects being studied as possible systemic treatments for this disease [12].

In summary, the rarity of this disease has limited our understanding of the behavior of and treatment options suitable for EHE. More prospective studies are necessary to better understand and manage this condition. Case presentations like in our patient and follow-up of these rare patients help expand the understanding of these patients and their disease behavior. It is necessary to report these cases to increase the number of available data for the future care of these rare patients.

## Conclusions

This patient is a prime example of how these rare EHE patients present to healthcare providers. Due to the rarity of these cases, continuous case reports are necessary to give practitioners a broader view of their findings and disease behavior. This allows for improved outcomes for EHE patients in the future. The biopsy result is usually unexpected and a full metastatic workup including local, brain, and chest scans are necessary. In this patient, the unfavorable location of the lesion margin led to the decision of debridement, followed by close observation of the lesion. The lesion in this patient may have a benign behavior therefore in this patient, the radiation to vital structures was deferred until some progression and follow-up are done to see if her EHE would continue to grow or stop. At that point, radiation treatment would be considered.
